# Relationship between dental caries, oral hygiene and malocclusion among Syrian refugee children and adolescents: a cross-sectional study

**DOI:** 10.1186/s12903-021-01993-3

**Published:** 2021-12-07

**Authors:** Nesreen A. Salim, Rasha A. Alamoush, Mariam Mohammad Al-Abdallah, Aya Ahmed Al-Asmar, Julian D. Satterthwaite

**Affiliations:** 1 Prosthodontic Department, School of Dentistry, The University of Jordan, Amman, 11942 Jordan; 2 Department of Paediatric Dentistry, Orthodontics, and Preventive Dentistry, School of Dentistry, The University of Jordan, Amman, Jordan; 3 Department of Conservative Dentistry, School of Dentistry, The University of Jordan, Amman, Jordan; 4 Division of Dentistry, School of Medical Sciences, University of Manchester, Oxford Road, Manchester, M13 9PL UK

**Keywords:** Dental caries, Malocclusion, Refugee, Oral health, Oral hygiene index, DMF index

## Abstract

**Background:**

Little is known about the oral disease burden in refugee children and associated risk factors. This cross-sectional study aimed to explore the oral hygiene status and prevalence of caries, and to investigate their association with malocclusion characteristics in a child refugee population.

**Methods:**

606 Syrian refugee children and adolescents aged 7–19 years, registered as refugees in Jordan and residing in Zaatari camp, were recruited to the study. Oral hygiene and caries status were recorded using DMFT (mean of decayed, missing, and filled permanent teeth) and OHI-S (Simplified Oral Hygiene Index) according to WHO criteria. Oral health results were then cross tabulated with previously reported malocclusion traits for the same study sample (crowding, spacing, contact point deflection and IOTN) to detect any associations. Statistical analysis was conducted using chi-square test, independent sample t-test, one-way ANOVA, Welch test and Post Hoc testing (Gabriel and Games-Howell).

**Results:**

Overall DMFT and OHI-S were 4.32 and 1.33 respectively with no difference between males and females. Around 40% of the sample showed ≥ 5 DMFT score. 96.1% of the sample either do not brush or brush occasionally: females showed better oral hygiene practices (*P* = 0.002). No significant differences in DMFT scores were noted for gender or age, other than the 7–9.9 year old group having significantly higher mean DMFT scores than all other age groups (*P* < 0.01);the mean of OHI-S was not significantly different between different age groups (*P* = 0.927). Subjects with malocclusion, specifically crowding, contact point deflection and IOTN grades 3, 4 and 5 had higher scores in both arches for OHI-S and DMFT than subjects without malocclusion traits, although this was not statistically significant for DMFT scores. Overall, patients with generalized spacing had a significantly lower OHI-S score than those without spacing (*P* = 0.021). Significant correlations were found between parameters of intra-arch and inter-arch relationships and oral health indices (DMFT and OHI-S).

**Conclusion:**

Malocclusion may increase the risk of caries and periodontal disease; the magnitude of this risk is amplified in populations with poor oral health and limited access to oral healthcare services, highlighting the need for preventive and curative oral health programs.

## Background

Malocclusion has been associated with psychosocial distress, discomfort, low quality of life, poor periodontal condition, and impaired masticatory function [1, 2]. This impacts quality of life and leads to increased demand for orthodontic treatment, especially for children and adolescents with this age group being susceptible to psychological trauma and influence on educational and social skills [3, 4]. The dental health component (DHC) of the Index of Orthodontic treatment Needs (IOTN) [5] aims to quantify treatment need based on the harmful effects of malocclusion: heterogeneous values of orthodontic treatment needs have been reported in different countries ranging from 71% in Jordanian school children to 93% in 11–14-year-old Italian children [6, 7]. Differences in ethnicity and study methodology underlie some of these variations [5].

Dental caries, along with periodontal disease, contributes significantly to the global burden of chronic diseases [8, 9], although the influence of malocclusion on disease levels is unclear, with conflicting results in relation to both periodontal condition [10, 11], and also caries [12–15]. Some traits of malocclusion might hamper efficient oral hygiene [16, 17]: for example, crowding is highly correlated to increased plaque indices and gingival inflammation [18] and caries may be associated with spacing [12], crowding [19], and increased or reduced overbite [12, 13]. Also, caries and early tooth loss may in turn lead to occlusal irregularities and malocclusion [20]. However, maintenance of good oral hygiene may be more important than the improvement provided by orthodontic treatment [10, 11].

Children and adolescents constitute about 35.2% of the total refugee population in Jordan [21] (nearly 330,000 below 17 years) and about 56.0% of the Zaatari camp population (over 43,000 below 17 years). Rates of dental disease have increased since the war in Syria began, and caries is the second most commonly reported health issue among refugees after the common cold [22], with the high prevalence of dental disease among immigrants and refugees being attributed to the difficulties in accessing dental services, cost, poor quality nutrition, poor diet and low priority given to dental care during the migration process [23–25].

The presence of malocclusion in refugee populations may further complicate their already-deteriorated oral health and negatively affect quality of life [26, 27]; however, the relationship between dental caries, oral hygiene and malocclusion has not yet been investigated in Syrian refugee children and adolescents and such information is important to target healthcare services and priorities according to an evidence-base. The aim of this study was to evaluate the overall oral health of refugee children to investigate any association with malocclusion traits. The specific objectives were to assess oral health using DMFT index and oral hygiene scores (OHI-S) and to evaluate any relationship to existing orthodontic problems.

## Methods

### Study design and study group

A cross-sectional clinical survey was conducted from October 2019 to December 2019. Participants were Syrian refugee children and adolescents aged 7–19 years, registered as refugees in Jordan and residing in Zaatari camp. All child attendees/parents to the camp dental clinic during this period were invited to participate.

#### Demographic and general dental data

Gender, age, and oral habits were recorded. Age was categorized into: A1: 7–9.9 yrs., A2: 10–12.9 yrs., A3: 13–15.9 yrs., A4: 16–19 yrs.

#### Clinical examination

Examination was carried out by a prosthodontist (first author) who had been previously trained in the standardized diagnostic criteria and the basic methods outlined by the WHO [28], assisted by 2 junior dentists. Prior to the main study, a group of 30 patients aged 7–19 years (not part of the study sample) were examined for intra-examiner calibration, with an excellent level of agreement (Cohen’s kappa coefficient = 0.94). A total of 606 individuals were examined in Zaatari dental clinics. The clinics had a high level of attendance, and there was thus no need for further active recruitment.

Sample size was calculated prior to data collection using expected sample size formula. Our target population were children and adolescents in Zaatari camp (around 37,000) and with a power of 85%, alpha value of 0.05 (a margin of error of 5%), and a confidence interval of 95% the calculated sample was 381 [29]. Therefore we aimed for a sample of around 600 to take into consideration that our study included multiple comparisons of proportions and ranked values.

Dental examinations were carried out on reclined patients using a basic disposable oral mirror and a WHO periodontal probe. The average time for examination time of each child was approximately 5 min. An oral health assessment form was developed, based on the model suggested by the WHO [28]; for each patient demographic (age, gender); frequency of tooth brushing was recorded, as well as clinical variables as below:

#### Dental caries

1. Inspection of caries prevalence and history was measured using DMFT (mean of decayed, missing, and filled permanent teeth).
2. Significant caries index (SiC) was obtained by calculating the mean DMFT/dmft score of the third of the population with the highest DMFT/dmft [30]. With SiC/dmft being the significant caries index for primary teeth and SiC/DMFT being the significance caries index for permanent teeth.

#### Oral hygiene

Oral hygiene status was registered using the oral hygiene index simplified (OHI-S) (a combination of the debris index and the dental calculus index to determine the status of oral hygiene). For those participants aged 5 to 6 years, labial surfaces of the 54, 64, 61, 82 and the lingual surface of 75 and 85 were assessed. For mixed dentitions the labial surface of 26 and the lingual surface of 46 were also considered. For participants with most of their permanent teeth the labial surfaces of 11, 26, 16, 31 and the lingual surfaces of 36 and 46 were examined [31].

### Statistical analysis

Data were assessed using SPSS version 23.0 (IBM Corp. Armonk, NY). Descriptive analysis and frequency tables were used for general description; a chi-square test was performed to identify any significant differences in the mean of the DMFT and OHI-S between gender and age groups. The mean of the DMFT and OHI-S was compared between genders using an independent sample t-test. To compare the DMFT and OHI between the four age groups as well as the five oral hygiene method groups, the homogeneity of variances was tested. For those proved homogeneous, one-way ANOVA was used, otherwise a Welch test was used. Significant differences between any of the tested groups were further investigated by Post Hoc testing (Gabriel and Games-Howell) to identify groups that were significantly different. The level of significance was set at *P* < 0.05.

The results of oral health were cross tabulated with malocclusion findings that have been reported previously for the same population sample (crowding, spacing IOTN and contact point deflection) [32].

## Results

### Descriptive statistics of the sample

606 children/adolescents participated in the study. 96.2% of the sample was fit and healthy and the remaining suffered from chronic disorders such as asthma, epilepsy and allergy. Demographics and oral hygiene practices are given in Table 1. The proportion with poor oral hygiene practices (did not brush) was significantly worse in males (*P* = 0.008), and those reporting good oral hygiene practices (brush once) was significantly higher in females (*P* = 0.002, Table 1).

**Table 1:** Age groups and oral health methods by gender

Parameters	Gender N (%)		Total (N = 606)	
	Male 280 (46.2)	Female 326 (53.8)	N	%
Age (years)				
A1 (7–9.9)	47 (17)	27 (8)	74	12.2
A2 (10–12.9)	141 (50)	181 (56)	322	53.1
A3 (13–15.9)	79 (28)	96 (29)	175	28.9
A4 (16–19)	13 (5)	22 (7)	35	5.8
Oral hygiene method				
None*	97 (35)	69 (21)	166	27.4
Rinse only	4 (1)	1 (0.3)	5	0.8
Irregular brush	118 (42)	130 (40)	248	40.9
Brush once a day*	22 (8)	64 (19.7)	86	14.2
Brush twice a day	39 (14)	62 (19)	101	16.7

N: Number of subjects

*Significance difference between males and females at *P* < 0.01

**Fig. 1:**
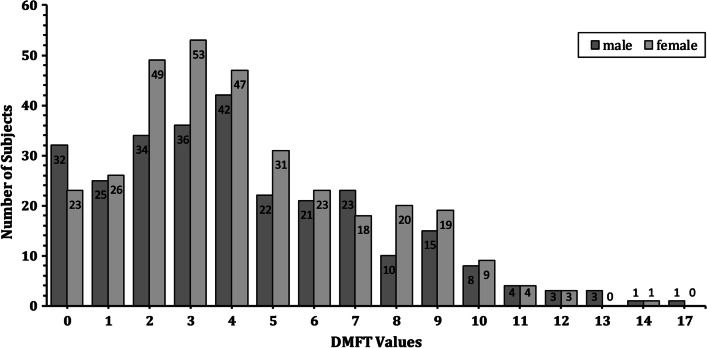
The range from 0 to 17 for DMFT count data of the study sample according to gender

The mean DMFT was 4.32 (4.33 for males and 4.31 for females); 92.4% of the subjects had a DMFT score > 0 and 39.4% of subjects had a DMFT ≥ 5.0 with a significant caries index (SiC) of 8 (Fig. 1). The mean of oral hygiene index simplified (OHI-S) was 1.33 (1.32 for males and 1.35 for females). No significant differences in DMFT were noted for gender or age, other than the 7–9.9 year old group having significantly higher mean DMFT than other age groups (*P* < 0.01).

Children with IOTN grade 3, 4 and 5 had significantly higher OHI-S than grade 1 (*P* < 0.01). The OHI-S in subjects with IOTN grade 1 was significantly lower than subjects with grade 3 and 4 (*P* = 0.031, *P* < 0.001 respectively: Table 2). Regression analysis showed that DMFT was not significantly correlated to the IOTN (*P* = 0.248), while OHI was significantly and positively correlated to the IOTN (*P* = 0.002).

**Table 2:** Comparison of DMFT and OHI-S means by IOTN group

Index on orthodontic treatment need (DHC)		I-Gender N (%)		Total (N = 606)	II-Overall OH indices	
		Males (N = 280)	Females (N = 326)	N (%)	DMFT Mean (SD)	OHI-S Mean (SD)
Grades and treatment needs						
Grade 1	No need	53 (19)	49 (15)	102 (16.8)	3.85 (2.55)	1.09^*α¥^ (0.57)
Grade 2	Little need	41 (15)	53 (16)	94 (15.5)	4.55 (3.08)	1.34 (0.72)
Grade 3	Borderline need	81 (29)	82 (25)	163 (26.9)	4.05 (2.90)	1.33* (0.76)
Grade 4	Definitive need	88 (31)	123 (38)	211 (34.8)	4.63 (3.29)	1.44^α^ (0.90)
Grade 5		17 (6)	19 (6)	36 (5.9)	4.39 (3.39)	1.47^¥^ (0.80)

^*^^α¥^Significant difference at P˂ 0.05 in the same column

OHI-S was significantly lower in those with no arch crowding compared to those with moderate and severe crowding (*P* = 0.001), and those with mild crowding had significantly lower OHI-S than those with severe crowding (*P* = 0.005). OHI-S was significantly and positively correlated to the severity of crowding in both arches (*P* < 0.001), and significantly and negatively correlated to the severity of spacing in the upper arch (*P* = 0.002) and in the lower arch (*P* = 0.031). OHI-S was not significantly different based on the severity of lower arch spacing although those with no upper arch spacing had significantly higher mean OHI-S than those with generalized spacing (*P* = 0.001). The DMFT was not significantly different based on the severity of upper or lower arch crowding (*P* = 0.153 and 0.158, respectively: Table four), although was significantly higher for those with generalized lower arch spacing than with no spacing. (*P* = 0.014). Overall, patients with generalized spacing had significantly lower OHI-S than patients with no spacing (*P* = 0.021) and patients with generalized spacing had significantly higher DMFT than patients with no spacing (*P* = 0.014, Table 3).

**Table 3:** Comparison of DMFT and OHI-S means by crowding and spacing

Parameters	Crowding				Spacing		
	None	Mild	Moderate	Severe	None	Localized	Generalized
Upper							
Mean (SD)							
DMFT	4.21 (3.05)	4.03 (2.75)	4.55 (3.25)	4.86 (3.27)	4.36 (3.00)	3.87 (2.76)	4.59 (3.61)
OHI-S	1.18*^α¥^ (0.64)	1.37* (0.78)	1.57^α^ (0.90)	1.48^¥^ (0.96)	1.39* (0.84)	1.28 (0.59)	1.09* (0.64)
Lower							
Mean (SD)							
DMFT	4.32 (3.11)	4.19 (2.95)	4.00 (2.92)	5.01 (3.25)	4.24* (2.98)	4.02 (2.91)	5.37* (3.69)
OHI-S	1.21*^¥^ (0.70)	1.27^α^(0.64)	1.48* (0.97)	1.71^¥α^ (1.03)	1.36 (0.81)	1.32 (0.66)	1.09 (0.62)

^*^^α¥^Significance differences at *P* < 0.05 in the same row for each parameter

In the upper arch, patients with no contact point deflection had significantly lower OHI-S than those with deflection of 1–2 mm and ˃ 4 mm (*P* = 0.007 and *P* = 0.004, respectively); those with deflection ˂ 1 mm had significantly lower OHI-S than other groups (*P* < 0.001, *P* = 0.002, *P* < 0.001 respectively). However in the lower arch, patients with no lower contact point deflection had significantly lower OHI-S compared to those with deflection of ˃4 mm (*P* = 0.004); those with lower arch contact point deflection of ˂ 1 mm had significantly lower OHI-S than those with deflection of 2–4 mm and ˃ 4 mm (*P* = 0.013 and *P* < 0.001, respectively). Patients with lower arch contact point deflection of 1–2 mm had significantly lower OHI than those with deflection of > 4 mm (*P* = 0.036, Table 4). OHI-S was significantly and positively correlated to the severity of contact point deflection in both arches (*P* < 0.001). Patients who brushed twice a day had significantly lower OHI-S scores compared to patients who reported no brushing (*P* < 0.001) and patients with irregular brushing habits (*P* < 0.001). DMFT scores showed no differences between all groups (*P* = 0.652, Table 5).

**Table 4:** Comparison of DMFT and OHI-S means by contact deflection

Deflection	DMFT Mean (SD)	OHI Mean (SD)
	*P* = 0.576	*P* = 0.000
Upper arch (n)		
None (149)	4.11 (2.67)	1.19 (0.63)*^α^
< 1 mm (84)	4.45 (3.35)	1.00 (0.53)^¥Ωψ^
1–2 mm (129)	4.10 (2.89)	1.48 (0.79)*^¥^
2–4 mm (118)	4.32 (2.84)	1.35 (0.76)^Ω^
> 4 mm (126)	4.68 (3.58)	1.56 (1.00)^αψ^

Deflection	DMFT Mean (SD)	OHI Mean (SD)
	*P* = 0.364	*P* = 0.000
Lower arch (N)		
None (127)	4.09 (2.84)	1.21 (0.73)*
< 1 mm (150)	4.22 (2.99)	1.16 (0.62)^αΩ^
1–2 mm (135)	4.10 (2.80)	1.31 (0.68)^¥^
2–4 mm (100)	4.67 (3.42)	1.48 (0.86)^α^
> 4 mm (94)	4.70 (3.32)	1.66 (1.02)*^Ω¥^

^*^^α ¥Ωψ^Significance differences at *P* < 0.05 in the same column for each parameter

**Table 5:** Comparison of DMFT and OHI-S means by oral hygiene (1 = none, 2 = rinse only, 3 = irregular brushing, 4 = brush once a day, 5 = brush twice a day)

Oral hygiene method groups	DMFT Mean (SD)	OHI-S Mean (SD)
	*P* = 0.652	*P* = 0.00
1 N = 166	4.48 (3.2)	1.48 (0.89)*
2 N = 5	3.20 (4.1)	1.73 (0.77)
3 N = 248	4.35 (3.0)	1.39 (0.78)^α^
4 N = 86	3.94 (2.5)	1.22 (0.70)
5 N = 101	4.34 (3.1)	1.02 (0.60)* ^α^

^*α^1 and 3 had significantly higher OHI-S than 5 at *P* < 0.01

## Discussion

The data concerning the extent of the oral health burden experienced by a growing number of refugees worldwide is limited [33]. Generally, oral health information about this underprivileged population suggests poor oral health and weak oral hygiene practices [22, 23]. To our knowledge, this is the first study to investigate the association between oral health status and malocclusion characteristics in child/adolescent refugees.

This study clearly demonstrated that the oral health status of child refugees in Zaatari camp (the second largest refugee camp worldwide) compares poorly with that of the general population and other child refugee populations, with very high unmet oral health needs [34–36]. Surprisingly, the oral health status of the refugee children in this study showed inferior oral health status compared to other Syrian refugees residing in districts outside the camp in the same country (Jordan); this could be because better overall services are provided outside the camp [37]. The prevalence of caries was 92.4%, which is a worryingly high percentage, justifying the need for efficient oral health programs to ensure that dental needs are targeted efficiently, appropriately and in a timely manner. Moreover, nearly 96% of this population showed none or very poor oral hygiene practices, which is a major factor to maintain adequate oral health, as highlighted by those with good oral hygiene practices showing significantly lower OHI-S scores.

Limited financial resources, scarcity of healthcare services, limited accessibility to proper nutrition and clean water, and being in a new community outside the habitual healthcare system, all are risk factors that increase oral health diseases and negatively affect the quality of life [22, 23, 38]. In addition, dental care is not frequently considered a main concern among refugees except for pain-associated emergencies as difficulty to access dental services is a considerable barrier [36]; subsequently, extraction is the most common treatment provided to child refugees, partly because teeth are severely destructed, and unexpectedly partly due to parental request—even for teeth that can be maintained [36]. This may reflect the lack of education and poor attitude towards the importance of dental treatment and the maintenance of optimal oral health [23]. Moreover, in this camp specifically, the dental services are funded by NGOs and depend on volunteer dentists, and are limited to emergency and routine dental treatment, including restorative care and extractions [23]. All expensive and sophisticated restorative, surgical, and prosthetic dental services are unavailable [23]. Long waiting times and a limited number of dental clinics are other challenging factors [39].

Although there was no significant difference in DMFT and OHI-S scores according to gender, females showed significantly better oral health practices than males concerning brushing frequency. These findings are consistent with previous studies [40, 41]. However, the negative impact of poor oral hygiene practices, especially in these males, may not manifest itself until adult life [12].

Caries prevalence was related to age, with 7–10 year-olds having the highest DMFT score. This could be related to the dental status at this age where most of primary teeth are carious or lost and many of permanent teeth have not yet erupted. Moreover, at this age the effect of malocclusion is very pronounced, in a growing arch with many erupting teeth and limited manual brushing efficiency compared to other older subjects: all these factors may affect the oral health of those subjects negatively [15].

Oral health status was significantly related to the orthodontic treatment need, especially for subjects with grade 4 or 5. The occlusal irregularities impede oral hygiene as well as self-cleansing mechanisms, thus result in increased accumulation of dental plaque and/or calculus [18, 41]. However, although the DMFT scores were higher in grades 3, 4 and 5, these differences were not significant. These results are in line with a previous study [12]. Although DMFT scores did not rise to a significant level, it indicates a trend toward more favorable dental health in subjects with low IOTN levels.

Crowding is an important occlusal trait that has been investigated extensively [2, 18, 41]. In the current study, a significant relation was detected between crowding in both arches and oral hygiene. Even moderate crowding showed higher OHI-S compared to mild or no crowding cases. Differences in methodologies and indices used to evaluate both occlusal and oral hygiene complicate comparisons with previous studies. A weak but significant relationship between occlusal crowding and increased gingivitis has been shown previously [2], additionally a strong association between crowding and gingival inflammation has been shown, however irregularities were not associated with significant gingival inflammation in objects with meticulous oral hygiene practices [18]. Conversely, another study showed no association between crowding and gingival condition [42]. In this study the mean of DMFT was not significantly different based on the severity of crowding in both arches, but all scores increased as the severity of crowding increased, which may indicate a negative impact of crowding on dental health and caries that may manifest in adult life [12]. Previous studies have indicated a significant correlation between crowding and caries [15, 43], while others have not shown this correlation [12].

Spacing has been studied as a favorable condition that improves cleansability and thus gingival heath. In this study, spacing in the upper arch was a favorable trait with lower OHI-S compared to subjects with no spacing. This is in line with a previous study, where subjects with spacing in the upper arch showed less gingivitis [41]. However, this was not the case for the lower arch, where the mean OHI-S was not significantly different based on the severity of lower arch spacing, although the scores were lower for subjects with spacing. This may be explained by poorer oral hygiene practices and food accumulation in the lower jaw, and the circumstantially disadvantageous location of lower molars which makes it more difficult to remove plaque from these teeth [44]. Accessibility and manual dexterity of brushing are other factors that may obscure the association between spacing and plaque accumulation in lower arch [41], for example higher DMFT scores in subjects with spacing in the lower arch compared to others could be due to food trapping and plaque accumulation and consequently more carious lesions. However, subjects with spacing had lower OHI-S scores than other subjects generally.

Oral health was highly related to the severity of the contact point deflection in both arches. Deflected contacts facilitate food impaction and hinder the effectiveness of oral hygiene practices with those having severe deflections having gingivitis and shallow periodontal pockets [18, 45]. This is also highlighted by improvement in gingival health with treatment of deflected incisors [46]. However, not all studies have shown a relationship between deflected contacts and number of plaque sites (in the presence of good oral hygiene) [42]. In this study, DMFT values were higher as the severity of deflection increased, and although this difference was not significant it highlights the negative impact of such irregularities on dental health and indicates an increased susceptibility to caries in the future, as has been confirmed by previous studies [15, 43].

## Limitations

The main limitation of the present study was that recording DMFT at a single point of time does not necessarily reflect the true oral health history, with previously carious teeth having been extracted. These factors may explain the high but not significant effect of malocclusion characteristics in this study on DMFT: additionally, caries can take a long time to manifest itself; all these could be contributing factors that weaken this association and perhaps explain the lack of increasing DMFT with age as might otherwise be expected.

The study population comprised a convenience sample from those who were attending dental clinics—the potential for bias exists with the possibility that those refugees who do not attend the dental clinics may have different disease rates or behavior. Despite these limitations, this study is the first to fill the gap in the literature by reporting on the oral health status of Syrian child refugees and their oral health needs, highlighting some of the challenges and malocclusion conditions that are experienced by this underprivileged population. These further complicate the oral health status in addition to factors such as limited facilities and accessibility to appropriate healthcare services. Maintaining meticulous oral hygiene measures is the gold standard to reduce the magnitude of the negative impact of malocclusion on oral health especially when orthodontic treatment is not available, as in the case for this population. This highlights the significance of implementing efficient targeted health promotion and preventive programs for this disadvantaged group.

## Conclusions

In the current study, inferior oral health status of Syrian refugee children/adolescents aged 7–19 years with high prevalence of dental caries was confirmed. Poor oral hygiene practices dominate the oral healthcare attitudes in the examined population, partly indicating the lack of dental services. Malocclusion is associated with oral hygiene and caries experience, which may justify the provision of orthodontic treatment for those children with severe malocclusion traits. Consequently, educational, preventive and curative programs should be implemented to improve child refugee dental care, especially those at high risk with severe malocclusion traits.

## Data Availability

All collected data from patients analyzed during this study are included in this published article. The datasets used and analyzed during the current study are available from the corresponding author on reasonable request.

## Abbreviations

DHC: Dental health component
DMFT/dmft: Mean of decayed, missing, and filled permanent teeth/ deciduous.
IOTN: Index of orthodontic treatment need
OHI-S: Simplified oral hygiene index
SD: Standard deviation
SiC: Significant caries index
WHO: World health organization
